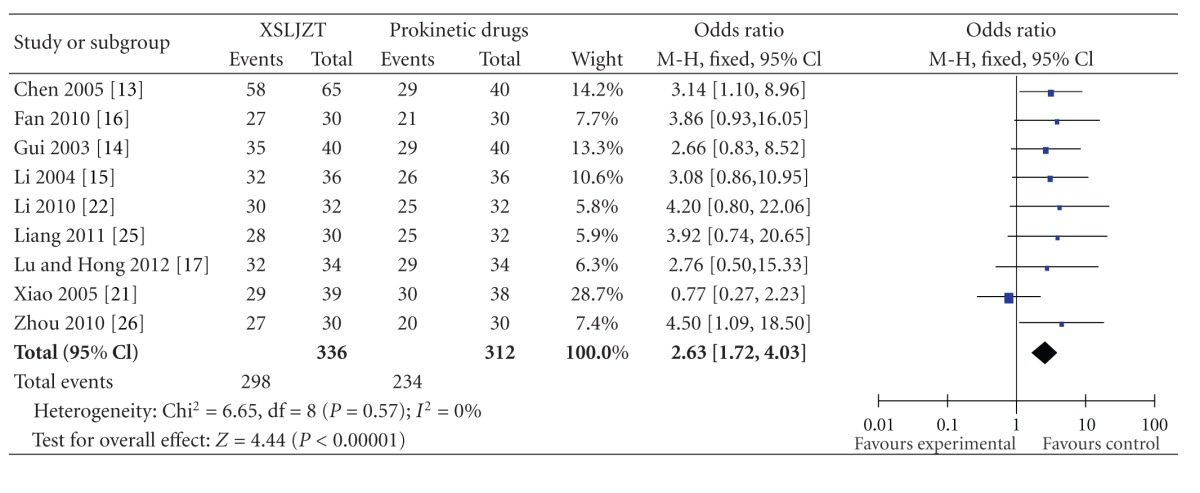# Chinese Herbal Medicine Liu Jun Zi Tang and Xiang Sha Liu Jun Zi Tang for Functional Dyspepsia: Meta-Analysis of Randomized Controlled Trials

**DOI:** 10.1155/2012/936459

**Published:** 2012-12-12

**Authors:** Ya Xiao, Yan-yan Liu, Ke-qiang Yu, Ming-zi Ouyang, Ren Luo, Xiao-shan Zhao

**Affiliations:** ^1^Department of Traditional Chinese Medicine, Southern Hospital, Southern Medical University, 1838 North Guangzhou Avenue, Guangdong, Guangzhou 510515, China; ^2^School of Traditional Chinese Medicine, Southern Medical University, 1838 North Guangzhou Avenue, Guangdong, Guangzhou 510515, China; ^3^Department of Science and Technology, Southern Medical University, 1838 North Guangzhou Avenue, Guangdong, Guangzhou 510515, China

## Abstract

*Objectives*. To assess the efficacy and safety of Liu Jun Zi Tang (LJZT) and Xiang Sha Liu Jun Zi Tang (XSLJZT) for treating functional dyspepsia. *Methods*. Literature searches were carried out on Medline database, Cochrane Library, CNKI database, Chinese Biomedical Literature database, Wanfang database, and VIP database up to July 2012. Hand search for further references was conducted. Study selection, data extraction, quality assessment, and data analyses were performed according to the Cochrane standards. *Results*. Fifteen publications in total were suitable for inclusion. There was evidence that LJZT compared with prokinetic drugs increased symptom improvement (odds ratio 1.96, 95% CI 1.15 to 3.36). There was also evidence that XSLJZT compared with prokinetic drugs increased symptom improvement (odds ratio 2.63, 95% CI 1.72 to 4.03). No adverse events were reported in LJZT or XSLJZT group in any of these randomized controlled trials. *Conclusion*. LJZT and XSLJZT might be more effective compared with prokinetic drugs in the treatment of functional dyspepsia, and no side effects are identified in the included trials. However, due to poor methodological quality in the majority of included studies, the potential benefit from LJZT and XSLJZT need to be confirmed in rigorously designed, multicentre, and large-scale trials.

## 1. Introduction

Functional dyspepsia, also known as nonulcer dyspepsia, is defined as persistent or recurrent abdominal pain or discomfort centred in the upper abdomen in the absence of an identifiable organic disease for at least a month [1–3]. The pathophysiological mechanisms that explain functional dyspepsia remain unclear; there is still no curative treatment available for patients with functional dyspepsia to date [4, 5]. A Cochrane review reported that prokinetics showed a significant relative risk reduction compared with placebo [6]. However, in spite of some beneficial effects, prokinetics have somewhat fallen into disgrace due to central nervous or cardiac adverse effects [7]. Bismuth salts, antacids, and sucralfate were of limited or even no benefit [8]. It seems, therefore, that development of new agents may be helpful in the therapy of patients with functional dyspepsia.

In traditional Chinese medicine (TCM), functional dyspepsia is considered nearly equivalent to the TCM term “stuffiness and fullness” [9], which is divided into different syndromes according to various clinical symptoms and signs. Spleen-deficiency syndrome is one of the basic syndromes. Liu Jun Zi Tang (LJZT), originated from the “Selected Stories of Traditional Chinese Medicine” in the Ming Dynasty, is a famous Chinese herbal formula that has been used for hundreds of years for the treatment of functional dyspepsia associated with the syndrome of “spleen deficiency” in China, Japan (named as “Rikkunshito” in Japanese), and other Asian areas. LJZT has been demonstrated to reduce chronic dyspepsia and produce significantly better gastric emptying than placebo in patients with functional dyspepsia [10]. The composition of LJZT includes the following eight Chinese herbs: ginseng, hoelen, atractylodes macrocephala koidz, liquorice root, pinelliae tuber, pericarpium citri, common ginger, and Jujube. In the Qing Dynasty, the classic book “TCM Prescriptions by Ancient and Modern Well-Known Physicians” originally described Xiang Sha Liu Jun Zi Tang (XSLJZT) which adds amomum villosum and costusroot with the function of promoting Qi circulation on the composition of LJZT [11]. Currently, It is noteworthy that in clinical practice, Qi-stagnation syndrome and spleen-deficiency syndrome often come together, which plays a key role in the treatment of functional dyspepsia. Chinese Society of Digestive Diseases, a branch of Chinese Association of Integrative Medicine, suggested that spleen-deficiency and qi-stagnation syndrome can be treated by XSLJZT [12].

How about the application of LJZT and XSLJZT in the treatment of functional dyspepsia and if there is difference in efficacy between the two formulas? With a view to answering these questions, the meta-analysis of randomized controlled trials evaluates the effects and safety of LJZT and XSLJZT in the treatment of functional dyspepsia as compared to the prokinetic agents.

## 2. Methods

### 2.1. Search Strategy

For meta-analysis, the following databases were searched: Medline database (1989 to July 2012), Cochrane Library (1993 to July 2012), CNKI database (1979 to July 2012), Chinese Biomedical Literature database (1990 to July 2012), Wanfang database (1982 to July 2012), and VIP database (1989 to July 2012), using MeSH headings: “functional dyspepsia,” “nonulcer dyspepsia,” “Liu Jun Zi Tang,” “Xiang Sha Liu Jun Zi,” “Rikkunshito,” “Rikkunshi-to,” and “Kosharikkunshito.” Papers published in English, Chinese, and Japanese were evaluated. Only studies available in the full paper were reviewed. A recursive manual search of cited references in published studies was performed to identify other relevant studies.

Studies were considered to be eligible for inclusion if they met all of the following criteria: (i) patients included in the study were diagnosed with functional dyspepsia; (ii) the study was performed as a randomized controlled trial (RCT); (iii) LJZT or XSLJZT was compared with prokinetic agents; (iv) criteria for successful treatment were clearly clarified, and treatment success was not measured in terms of the scores of illness severity or the intensity of individual symptoms; (v) treatment lasted for 2 weeks or more.

For the meta-analysis, the terms of “patients without symptoms,” “patients with significant improvement of symptoms,” “patients with excellent or good results,” and equivalent expressions were considered to be “successful treatment.”

### 2.2. Data Abstraction

Two researchers abstracted the data from the studies independently to avoid bias in the data abstraction process. All data were checked for internal consistency, and disagreements were resolved by discussion between the two investigators. Details abstracted from the studies included the name of the first author, location of the report, year of publication, age of subjects in the LJZT or XSLJZT arm and the control arm, male-female ratio in each arm, number of participants in each arm, and duration of treatment.

### 2.3. Data Analysis

Meta-analysis was carried out using Review Manager software (version 5.0.18 2009), provided by the Cochrane Collaboration. Dichotomous data were presented as odds ratios (ORs) and continuous outcomes as mean difference (MD), both with 95% confidence interval (CI). The chi-squared test for heterogeneity was performed, and heterogeneity was presented as significant when *I* ^2^ is over 50% or *P* < 0.1. Random effect model was used for the meta-analysis if there was significant heterogeneity, and fixed effect model was used when the heterogeneity was not significant.

## 3. Results

### 3.1. Description of Included Trials

**Figure 1:**
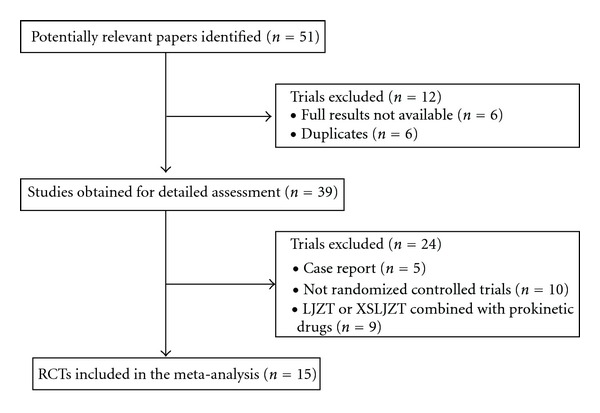
Study selection process.

A total of 51 publications were identified by both computer search and manual search of cited references. 45 articles were retrieved in full text, while 6 references were published in duplicate. After further reading, we excluded 5 case reports, 10 no-randomized controlled trials, and 9 studies having the treatment of LJZT or XSLJZT combined with prokinetic drugs. Finally, a total of 15 studies were included in the meta-analysis.  Figure 1 is a flow chart of the study selection process.

All 15 publications included were of a randomization procedure, single center. 14 studies were published in Chinese and the other one in English. Numbers of participants of the individual studies varied from 27 to 160 with a total of 1149 participants included in this paper. The participants were at different ages, ranging from 18 to 70, 606 of whom were females and 543 were males (Table 1).

Eight of the studies used domperidone (also called Motilium) in the control group [13–20]. In four studies, cisapride was used as the control [21–24]. In remaining studies, Mosapride was the control [25–27]. Only one study lasted 2 weeks, and the remaining 14 studies had a duration of 4 weeks. The interventions were given orally. Four classes were used to evaluate treatment effects including cure, significant effective, effective, and ineffective according to the scores reducing rate.

### 3.2. Methodological Quality of Included Trials

The Jadad scale is a 5-point scale for assessing the quality of randomized trials in which three points or more indicate superior quality [28]. The scale contains two questions each for randomization procedure and blinding and one question evaluating reporting of withdrawals and dropouts. Of the 15 RCTs, two trials were of superior quality according to the Jadad score (≥3 points) [16, 17]. All studies described the randomization procedure, but none of them mentioned allocation concealment. One of the studies described blinding of participants [17]. Two studies reported the dropouts information and mentioned followup, but did not use intention to treat analysis [16, 20]. Among all trials, the characteristics of subjects in different treatment groups were similar at baseline (age, sex, race, and disease course).

### 3.3. Liu Jun Zi Tang versus Prokinetic Drugs

#### 3.3.1. Effect Rate

Five randomized controlled trials evaluated LJZT compared to prokinetic drugs. All the studies were published in Chinese. Odds ratio was used to assess the change of dyspeptic symptoms in patients with functional dyspepsia.  Table 2 shows the OR and summary OR for each trial. The summary OR was 1.96 (95% CI: 1.15–3.36), indicating that functional dyspepsia patients with LJZT treatment had almost 2-fold higher probability of symptom relief compared with those with prokinetic drugs.

#### 3.3.2. Acylated Ghrelin

One trial which was performed in Japan provided data of plasma acylated ghrelin [20]. The baseline of acylated ghrelin had no significant difference between two groups. LJZT treatment had a tendency to increase acylated ghrelin level as compared with prokinetic drugs group at the end of the study (15.6 ± 16.0 versus 6.6 ± 6.1 fmol/mL), but this effect failed to reach statistical significance (WMD, 9; 95% CI, −0.27 to 18.27; *P* = 0.06) (Table 3). 

### 3.4. Xiang Sha Liu Jun Zi Tang versus Prokinetic Drugs

#### 3.4.1. Effect Rate

A total of nine randomized controlled trials tested XSLJZT compared to prokinetic drugs. The studies were all performed in China. The duration of treatment was 4 weeks. Eight trials reported effects in favor of XSLJZT compared with prokinetic agents at the end of treatment [13–17, 22, 25, 26], while one trial reported negative result [21]. A meta-analysis of the nine trials (*n* = 648) showed a significant increase of symptom improvement over prokinetic drugs (odds ratios 2.63 points, 95% CI 1.72–4.03). The chi-squared test for heterogeneity signified homogeneity among the nine studies (*P* = 0.57;  *I* ^2^ = 0%) (Table 4). However, the number of studies (*n* = 9) was improper to produce a meaningful funnel plot.

### 3.5. Safety

Of the 15 RCTs, twelve trials did not mention the occurrence of adverse events. Fan [16] and Arai et al. [20] reported no side effect in LJZT or XSLJZT group and prokinetic drugs group during treatment. Tian and Wang [27] reported that 16 out of 80 patients had experienced a variety of symptoms including abdominal pain, diarrhea, rugitus, and loose stools in the prokinetic drugs group. These symptoms could be tolerated by patients. No adverse events were reported in LJZT or XSLJZT group in any of these randomized controlled trials.

## 4. Discussion

Based on the meta-analysis of 15 randomized controlled trials, it can be documented that both Xiang Sha Liu Jun Zi Tang and Liu Jun Zi Tang are more effective than prokinetic drugs for patients suffering from functional dyspepsia. Interestingly, we found that the number of RCTs about XSLJZT in the treatment of functional dyspepsia was more than LJZT, and XSLJZT seemed to show a more significant increase of symptom improvement over prokinetic drugs as compared to LJZT (odds ratios 2.63 versus odds ratios 1.96).

Experimental data has demonstrated that LJZT may be thought to improve functional dyspepsia by reversing the existing impaired adaptive relaxation, leading to an improvement in delayed gastric emptying and stimulation of ghrelin secretion [29–31]. XSLJZT can significantly improve electrogastrogram, promote gastrointestinal motility and gastric emptying, decrease gastric sensitivity, and regulate gastrointestinal hormone [32–35]. The effectiveness of LJZT and XSLJZT in the treatment of functional dyspepsia might be due to different functions of the single plant extracts. Pharmacological studies have shown that ginseng radix and pinelliae tuber can accelerate gastrointestinal motility and cause increases in intestinal propulsion [36, 37]. Hoelen could be applicable for the treatment of functional dyspepsia by regulating gastric movement through its facilitatory effect on efferent discharge of the gastric vagus nerve [38]. Atractylodes macrocephala koidz can improve the delayed gastric emptying and promote gastrointestinal motility [39]. Isoliquiritigenin plays a dual role in regulating gastrointestinal motility, both spasmogenic and spasmolytic [40]. Costusroot has been demonstrated to shorten the time of gastric emptying and increase plasma motilin concentration [41]. All of these studies' results suggest that LJZT and XSLJZT can produce a potential effect of multitarget therapy, which seems appropriate in the treatment of functional dyspepsia caused by multiple factors.

The safety profile of LJZT and XSLJZT appears satisfactory, both in view of the data derived from clinical and observational studies and considering the fact that the drugs have been prescribed for patients with functional dyspepsia for hundreds of years in China. The explanation of the encouraging safety might be that the multitarget effect of herbal preparations may produce relatively low concentration of active ingredients.

In this meta-analysis, there were several limitations. No multicenter, large sample, and cooperative studies were found, and most of the trials were of small sample size, yet no studies estimated the sample size. Among all 15 articles, 2 applied random number tables [16, 17]; the remaining 13 studies did not provide the method of randomization procedure. Due to inadequate reporting of the allocation sequence, we cannot draw a judgement of whether or not they had been conducted properly. And inadequate reporting of allocation concealment, blinding, dropouts account, and intention to treat analysis in all the studies may have created potential selection biases, performance biases, and detection biases. Although we conducted comprehensive searches and tried to avoid bias, since most of the trials were published in Chinese, there remained the possible existence of publication bias.

In conclusion, the results of the present paper preliminarily confirm that LJZT and XSLJZT are more effective compared with prokinetic drugs in the treatment of functional dyspepsia. However, the general quality of the reports was moderate to low, and most of the trials were published in Chinese. Therefore, the effectiveness of LJZT and XSLJZT needs further evidence of rigorously designed, multicentre, large-scale, and transnational cooperative RCTs to prove, which would contribute greatly to the treatment of functional dyspepsia worldwide.

## Figures and Tables

**Table 1:** Randomized controlled trials of LJZT or XSLJZT for functional dyspepsia.

Study ID	Year	Participants	Mean age (years)	Interventions		Treatment duration	Symptom improvement	Jadad score
				Experimental	Control (prokinetic drugs)			
Chen [13]	2005	105 people (38 men and 67 women)	45.7	XSLJZT (BID)	Domperidone (10 mg, TID)	4 weeks	(1) TER: 89.2% (58/65) (2) TER: 72.5% (29/40)	1
Xiao [21]	2005	77 people (33 men and 44 women)	43.0	XSLJZT (BID)	Cisapride (5 mg, TID)	4 weeks	(1) TER: 74.4% (29/39) (2) TER: 78.9% (30/38)	1
Gui [14]	2003	80 people (35 men and 45 women)	46.1	XSLJZT (BID)	Domperidone (10 mg, TID)	4 weeks	(1) TER: 87.5% (35/40) (2) TER: 72.5% (29/40)	2
Li [15]	2004	72 people (27 men and 45 women)	47.1	XSLJZT (BID)	Domperidone (10 mg, TID)	4 weeks	(1) TER: 88.8% (32/36) (2) TER: 72.2% (26/36)	2
Liang [25]	2011	62 people (28 men and 34 women)	38.0	XSLJZT (BID)	Mosapride (5 mg, TID)	4 weeks	(1) TER: 93.3% (28/30) (2) TER: 78.1% (25/32)	1
Zhou [26]	2010	60 people (29 men and 31 women)	35.9	XSLJZT (BID)	Mosapride (5 mg, TID)	4 weeks	(1) TER: 90.0% (27/30) (2) TER: 66.7% (20/30)	2
Li [22]	2010	64 people (35 men and 29 women)	45.3	XSLJZT (BID)	Cisapride (5 mg, TID)	4 weeks	(1) TER: 93.8% (30/32) (2) TER: 78.1% (25/32)	1
Fan [16]	2010	60 people (29 men and 31 women)	48.5	XSLJZT (BID)	Domperidone (10 mg, TID)	4 weeks	(1) TER: 90.0% (27/30) (2) TER: 70.0% (21/30)	3
Lu and Hong [17]	2012	68 people (40 men and 28 women)	42.5	XSLJZT (BID)	Domperidone (10 mg, TID)	4 weeks	(1) TER: 94.1% (32/34) (2) TER: 85.3% (29/34)	3
Zhang and Shun [23]	2002	64 people (21 men and 43 women)	44.3	LJZT (BID)	Cisapride (5 mg, TID)	4 weeks	(1) TER: 93.9% (31/33) (2) TER: 93.6% (29/31)	2
Tian and Wang [27]	2005	160 people (73 men and 87 women)	34.4	LJZT (BID)	Mosapride (5 mg, TID)	2 weeks	(1) TER: 85.0% (68/80) (2) TER: 87.5% (70/80)	2
Chen and Zhang [18]	2008	80 people (37 men and 43 women)	32.3	LJZT (BID)	Domperidone (10 mg, TID)	4 weeks	(1) TER: 90.0% (36/40) (2) TER: 75.0% (30/40)	1
Wang [19]	2010	110 people (56 men and 54 women)	42.1	LJZT (BID)	Domperidone (10 mg, TID)	4 weeks	(1) TER: 91.7% (55/60) (2) TER: 74.0% (37/50)	1
Zhao and Che [24]	2009	60 people (46 men and 14 women)	52.0	LJZT (BID)	Cisapride (5 mg, TID)	4 weeks	(1) TER: 96.7% (29/30) (2) TER: 80.0% (24/30)	1
Arai et al. [20]	2012	27 people (16 men and 11 women)	57.8	LJZT (TID)	Domperidone (10 mg, TID)	4 weeks	Not mentioned	2

LJZT: Liu Jun Zi Tang; XSLJZT: Xiang Sha Liu Jun Zi Tang; TER: total effective rate; QD: once a day; BID: twice a day; TID: three times a day.

**Table 2:** Odds ratios (ORs) and summary OR for Liu Jun Zi Tang (LJZT) trials. OR and their 95% confidence intervals are presented with weighting in a fixed effect model. OR > 1.0 indicates that the symptomatic improvement of functional dyspepsia is higher in the LJZT group than that in prokinetic drugs group.

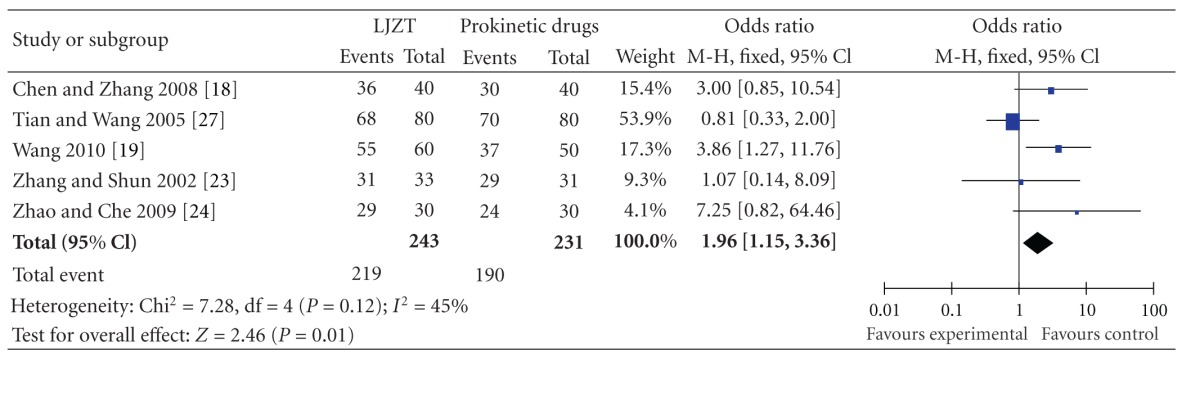

**Table 3:** Mean difference (MD) for Liu Jun Zi Tang (LJZT) trials. MD and their 95% confidence intervals are presented with weighting in a fixed effect model. There was no statistically significant difference between two groups in plasma acylated ghrelin (WMD, 9; 95% CI, −0.27 to 18.27; *P* = 0.06).

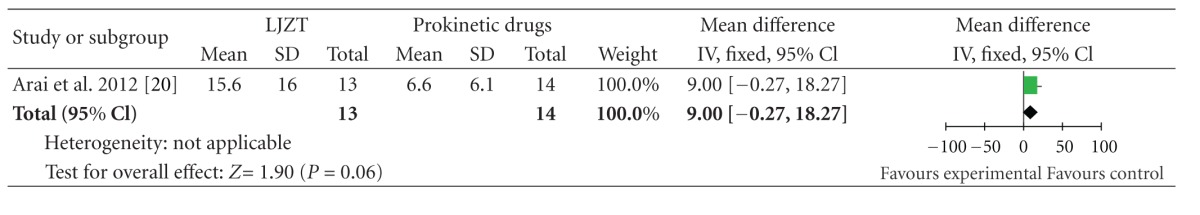

**Table 4:** Odds ratios (OR) and summary OR for Xiang Sha Liu Jun Zi Tang (XSLJZT) trials. OR and their 95% confidence intervals are presented with weighting in a fixed effect model. OR > 1.0 indicates that the symptomatic improvement of functional dyspepsia is higher in the XSLJZT group than that in prokinetic drugs group.